# Relationship of parental health-related behaviours and physical fitness in girls and boys

**DOI:** 10.1007/s10389-014-0636-5

**Published:** 2014-07-22

**Authors:** Nanette Erkelenz, Anja C. Schreiber, Susanne Kobel, Sarah Kettner, Clemens Drenowatz, Jürgen M. Steinacker

**Affiliations:** Division of Sports and Rehabilitation Medicine, Department of Internal Medicine II, Ulm University Medical Centre, Frauensteige 6, 89075 Ulm, Germany

**Keywords:** Exercise, Body weight, Children, Home environment

## Abstract

**Aim:**

Physical activity (PA) and physical fitness (PF) are known to be closely connected. Various environmental and biological constraints have been shown to influence children’s PA with parents being among strong determinants of their children’s PA behaviour. However, little is known about parental influence on PF in children. Therefore, the purpose of this study was to identify the influence of parental health-related behaviours and attitudes on PF in boys and girls.

**Subjects and methods:**

Baseline data of 1,875 primary school children (7.1 ± 0.6 years; 50 % male) were included in the analyses. Lateral jumping performance was used as a proxy for whole-body coordination and the 6-min run for cardiovascular fitness. Parental health-related behaviours, attitudes and sociodemographic variables were assessed via questionnaire. Regression analyses, adjusting for age and BMI, were performed separately for boys and girls.

**Results:**

The final models of the regression analyses showed that children’s age and BMI are significantly related to PF. Mothers’ self-efficacy to encourage their children to be active is significantly associated with boys’ coordination and cardiovascular fitness and girls’ coordination. Mothers’ PA affects PF in boys, not in girls. Maternal smoking has a significantly negative effect on both boys’ and girls’ cardiovascular fitness.

**Conclusion:**

This study shows that parental health-related behaviours and self-efficacy to encourage their children to be active affect children’s PF. Influencing factors, however, differ in girls and boys, and mothers seem especially influential.

## Introduction

It is well documented that sufficient physical activity (PA) and moderate to high levels of cardiovascular fitness are essential for childhood development and have numerous health benefits in children and adolescents (Ekelund et al. 2007). PA during childhood and adolescence is beneficially associated with body fatness and well-being at young adult age (Boreham and Riddoch 2001). For children, at least 60 min of moderate to vigorous PA (MVPA) daily are recommended (WHO 2010). In order to increase cardiovascular fitness even more time in moderate to vigorous intensity activity is necessary (Andersen et al. 2006). Nevertheless, a considerable number of children currently do not meet these recommendations (Ekelund et al. 2011). Low PA levels during childhood are particularly alarming since the basis of adequate PA levels and an active lifestyle in adulthood are established during early childhood (Baker et al. 2007).

Various environmental and biological factors have been shown to influence children’s PA (Kettner et al. 2012), and parents have been suggested to play a crucial role (Gustafson and Rhodes 2006). Results from studies on associations between parental activity behaviour and children’s PA, however, have been equivocal (Gustafson and Rhodes 2006; Trost and Loprinzi 2011). It seems that parental PA is not necessarily key for children’s PA, whereas parental support and encouragement were found to be more important than being an active role model (Trost et al. 2003).

PA and physical fitness (PF) are known to be closely connected, whilst an increase in PA mostly leads to increases in PF (Dencker et al. 2006). Therefore, not only children’s PA is influenced by biological and environmental factors; they also appear to play an important role in children’s PF levels (Ortega et al. 2008). Additionally, PF, especially cardiovascular fitness, is known to have favourable effects on health in children and adolescents (Ekelund et al. 2007). In a large-scale European study of children and adolescents an independent association between cardiorespiratory fitness and PA with metabolic risk factors has been shown (Ekelund et al. 2007).

At this time, little is known about parental influence on children’s PF as previous studies mainly focussed on parental PA and children’s PF (Cleland et al. 2005; Martín-Matillas et al. 2012). Overall, these studies showed a positive relationship between parental PA and children’s PF. An early study analysed the influence of parental encouragement and attitudes on children’s PF (McMurray et al. 1993) but found no associations. Recent research, however, has shown an association between paternal encouragement and adolescents’ cardiorespiratory fitness (Martín-Matillas et al. 2012).

Whilst parental PA has been shown to be associated with cardiorespiratory fitness in children (Cleland et al. 2005), there is hardly any research focusing on parental health-related behaviours and their confidence to encourage their children to be physically active and the effects this has on boys’ and girls’ PF. Therefore, the purpose of this study was to identify the influence of parental health-related behaviours and self-efficacyto encourage PA on PF in boys and girls.

## Methods

### Design and participants

Baseline data of 1,875 primary school children (7.1 ± 0.6 years; 50 % male) participating in the school-based health-promotion programme “Join the Healthy Boat” were included in the analyses. Details about the programme and the evaluation study have already been published elsewhere (Dreyhaupt et al. 2012). Parents’ written, informed consent as well as child assent was obtained prior to data collection. The study was approved by the Ministry of Culture and Education and the university’s ethics committee and is in accordance with the Declaration of Helsinki.

### Anthropometric measures

Children’s height (cm) and body mass (kg) were taken by trained technicians during a school visit. Following standardised procedures, height was measured to the nearest 0.1 cm using a stadiometer (Seca 213, weighing and measurement system, Hamburg, Germany) and weight was measured to the nearest 0.05 kg using calibrated electronic scales (Seca 862, weighing and measurement systems, Hamburg, Germany). Subsequently, body mass index (BMI) was calculated by dividing children’s weight (kg) by their height (m) squared. To determine children’s weight status, their BMI was converted to BMI percentiles (BMIPCT) using German reference data (Kromeyer-Hauschild et al. 2001). According to recommendations (AGA) (Kromeyer-Hauschild et al. 2001; AGA = German work group for obesity in childhood and adolescence), children with BMIPCTs between 90 and 97 were classified as overweight, with BMIPCTs of more than 97 as obese. Parental weight status (BMI) was calculated based on self-reported height and weight.

### Physical fitness

Two components of the Dordel Koch Test (Graf et al. 2004), which was performed during a school visit, were used as predictors for children’s PF. Children performed two trails of 15 s lateral jumping with the sum of both trails being used as proxy for whole body coordination and muscular endurance. A single trail for distance covered during a 6-min run was used as an indicator for cardiovascular fitness. Both subtests have been standardised and validated.

### Demographics

Family level of education was assessed according to CASMIN classification (Brauns et al. 2003) and then dichotomised into tertiary and elementary/intermediatelevel of education, considering the highest education of either parent. Migration status was determined based on parental place of birth (outside of Germany) or the language spoken during the child’s first years of life (other than German). Household earnings were assessed on a seven-pointscale and subsequently dichotomised into low income (up to 1,750 €) and medium and high income (above 1,750 € per month).

### Parental health-related behaviours and self-efficacy

Parental health-related behaviours and self-efficacy were assessed via parental questionnaire. Parents were asked whether they classify themselves as physically active or not (yes or no). In another study we found that the self-classification corresponded to reported time spent in organised sports (Erkelenz et al. 2014). Smoking habits were determined using a three-point scale (no, ex-smoker, yes) and categorised into current smokers/non-smokers. Self-efficacy refers to parental confidence in their ability to ensure a sufficient level of PA and was assessed using a four-pointscale (I am able to ensure my child is sufficiently physical active) and subsequently dichotomised by the median (able to ensure a sufficient level of PA and not able to ensure a sufficient level of PA).

## Statistics

Descriptive statistics including means and standard deviations (SD) or prevalence were calculated. Gender differences and group differences regarding migration and smoking habits were analysed using Student’s t-tests and correlation analyses were used to identify factors associated with children’s PF. Identified factors were then included in multivariate regression analyses with backward elimination to verify associated factors. Regression analyses for 6-min run performance and lateral jumps were computed for girls and boys separately, using age and BMI as covariates. Due to missing values sample sizes may vary for different analyses. All data analyses were conducted using PASW 19.0.01.

## Results

### Participants’ characteristics and motor skills

Sample characteristics are shown in Table 1. There was no difference in age, BMI and weight status between boys and girls. Lateral jumping performance did not differ either, but boys displayed significantly better results on the 6-min run [t(1856) = 8.9, *p* < 0.001].

**Table 1:** Participants’ characteristics

	All	Boys	Girls
Participant [n (%)]	1875 (100)	964 (51.4)	911 (48.6)
Age [years (SD)]	7.1 (0.6)	7.1 (0.6)	7.0 (0.6)
BMI [kg/m^2^ (SD)]	16.0 (2.2)	16.1 (2.2)	16.0 (2.2)
Overweight [n (%)]	101 (5.4)	51 (5.3)	50 (5.5)
Obesity [n (%)]	82 (4.3)	49 (5.1)	33 (3.5)
6-min run performance [m (SD)]	846.8 (121.9)	870.6 (127.9)	821.6 (109.8)
Lateral jumps [n (SD)]	41.4 (12.7)	41.0 (12.5)	41.8 (12.9)

### Health-related behaviours and self-efficacy

Parental characteristics regarding the studied variables are shown in Table 2. Almost one third of the mothers and two thirds of the fathers were classified as overweight/obese. There was no sex difference in parental characteristics between boys and girls except for parental self-efficacy to encourage their children to be sufficiently active, which was higher in boys [χ2 (1) = 6.12, *p* < 0.05].

**Table 2:** Prevalence of parental responses to studied variables

	N	Boys	Girls
Overweight mother, kg/m^2^ > 25 [n (%)]	1,522	237 (30.5)	233 (31.2)
Overweight father, kg/m^2^ > 25 [n (%)]	1,422	429 (59.0)	431 (62.0)
Migration background [n (%)]	1,549	248 (31.1)	254 (32.3)
Low family income < 1750 € [n (%)]	1,435	97 (13.3)	100 (14.1)
Tertiary Education level [n (%)]	1,054	254 (32.1)	251 (32.7)
Mothers with high confidence in their ability to ensure a sufficient level of child’s PA [n (%)]	1,577	455 (56.9)	395 (50.8)
Fathers with high confidence in their ability to ensure a sufficient level of child’s PA [n (%)]	1,463	419 (56.2)	324 (45.1)
Mothers smoking [n (%)]	1,595	164 (20.4)	165 (20.8)
Fathers smoking [n (%)]	1,529	228 (29.5)	227 (30.0)
Physically active mothers [n (%)]	1,527	446 (57.7)	456 (60.5)
Physically active fathers [n (%)]	1,446	415 (56.5)	415 (58.3)

In preliminary analyses using t-testsfor dichotomised independent variables and correlations for continuous variables, possible factors related to PF in girls and boys were identified (see table 3 and 4). Variables with significant associations (*p* < 0.05) with PF were included in the subsequent regression analyses.

**Table 3:** Possible factors related to endurance (6-min run) and coordination (lateral jumps) in boys and girls

	Boys		Girls	
	Lateral jumps (mean ± SD)	6-min run (mean ± SD)	Lateral jumps (mean ± SD)	6-min run (mean ± SD)
Mothers’ confidence in their ability to ensure a sufficient level of child’s PA				
High	43.14 ± 13.02***	890.05 ± 125.69***	43.43 ± 13.24**	831.83 ± 104.21
Low	39.24 ± 11.46	5858.22 ± 123.9	40.47 ± 12.58	3.22 819.64 ± 11
Fathers’ confidence in their ability to ensure a sufficient level of child’s PA				
High	43.20 ± 13.04**	888.99 ± 125.98*	43.60 ± 13.38*	833.11 ± 107.81
Low	40.10 ± 13.04	867.89 ± 120.43	40.76 ± 12.82	820.58 ± 111.16
Mothers’ PA				
Yes	42.55 ± 13.08**	888.7 ± 129.26**	42.81 ± 13.08*	837.15 ± 107.39***
No	40.25 ± 11.39	863.71 ± 120.09	40.83 ± 12.98	807.19 ± 109.32
Fathers’ PA				
Yes	42.67 ± 13.01*	893.79 ± 120.79**	42.16 ± 12.96	832.27 ± 107.13
No	40.57 ± 11.72	862.24 ± 126.90	42.01 ± 13.37	118.06 ± 111.54
Mothers’ smoking				
Yes	40.40 ± 12.46	836.13 ± 137.89***	40.97 ± 12.48	790.83 ± 103.07***
No	41.66 ± 12.48	885.1 ± 121.7	42.05 ± 13.20	833.84 ± 107.38
Fathers’ smoking				
Yes	40.72 ± 12.81	859.76 ± 128.66*	1.89 ± 13.57	808.18 ± 100.26**
No	41.65 ± 12.39	883.67 ± 123.89	42.09 ± 13.08	832.71 ± 111.40
Parental education level				
High	41.69 ± 13.76	885.29 ± 120.19	44.27 ± 12.60**	842.05 ± 109.85**
Low	41.40 ± 11.94	875.21 ± 127.28	40.83 ± 13.04	819.04 ± 105.85
Family income				
High	42.04 ± 12.46*	887.55 ± 122.50***	42.15 ± 13.16	826.89 ± 106.90**
Low	38.86 ± 12.30	837.81 ± 132.55	1.24 ± 13.52	799.77 ± 113.48
Migration				
Yes	41.42 ± 11.84	860.56 ± 126.89*	41.74 ± 13.70	799.15 ± 109.49***
No	41.57 ± 12.84	882.55 ± 126.26	2.11 ± 12.88	837.07 ± 105.66

*p < 0.05; **p < 0.01; ***p < 0.001

**Table 4:** Correlations of children’s age, BMI and parental BMI with endurance (6-min run) and coordination (lateral jumps)

	Boys		Girls	
	Lateral jumps	6-min run	Lateral jumps	6-min run
Age	0.39**	0.16**	0.36***	0.22**
BMI	−0.11**	−0.24**	−0.09**	−0.28**
BMI fathers	−0.03	−0.04	−0.01	−0.14**
BMI mothers	−0.06	−0.09*	−0.05	−0.15**

*p < 0.05; **p < 0.01

Tables 5 and 6 show the final models for girls and boys. Children’s age and BMI are closely related to their PF. Mothers’ confidence in their ability to encourage their children to be sufficiently active is associated with both components of PF, coordination and cardiovascular fitness in boys. In girls, mothers’ confidence in their ability to encourage their children to be sufficiently active was only associated with coordination. High levels of maternal PA were positively related to PF in boys but not in girls. Furthermore, maternal smoking was associated with lower cardiovascular fitness in girls and boys. There was no association between parental characteristics and children’s PF. In addition, high family income was associated with boys’ coordination and girls’ cardiovascular fitness and high parental education affected girls’ coordination. Further, non-migration was associated with cardiovascular fitness in girls.

**Table 5:** Final regression model for factors associated with coordination (lateral jumps) and endurance (6 min run) in boys

	Lateral jumps		6-min run	
	B	95 % CI	B	95 % CI
Constant	−15.96**	[−27.82,− 4.09]	769.71**	[6661.25, 878.17]
Age	8.45**	[7.05, 9.86]	54.75**	[40.98, 68.52]
BMI	−0.57*	[−1.01, −0.13]	−18.48**	[−22.79, −14.18]
Mothers’ confidence in their ability to ensure a sufficient level of child’s PA	4.19**	[2.47,5.92]	21.60*	[4.64, 38.57]
Mothers’ PA	2.16*	[.43, 3.89]	17.87*	[.77, 34.96]
Family income	3.59**	[1.00, 6.17]		
Mothers’ smoking			−38.15**	[−59.94, −16.35]
R^2^	0.21		0.16	
F	35.04**		28.70**	
N	673		735	

*CI* confidence interval; *p < 0.05; **p < 0.01

**Table 6:** Final regression model for factors associated with coordination (lateral jumps) and endurance (6 min run) in girls

	Lateral jumps		6-min run	
	B	95 % CI	B	95 % CI
Constant	−9.58	[−20.50,− 1.34]	750.02**	[643.78, 856.26]
Age	8.91**	[7.55, 10.26]	39.84**	[27.12, 52.55]
BMI	−0.85*	[−1.25, −0.45]	−12.74**	[−16.26, −9.22]
Mothers’ confidence in their ability to ensure a sufficient level of child’s PA	2.66**	[0.99, 4.32]		[4.64, 38.57]
Family income			11.19	[−12.60, 34.98]
Mothers’ smoking			−25.51*	[−44.47, −5.94]
Parental education level	3.02**	[1.25, 4.79]		
Migration			−29.54**	[−46.49, −12.56]
R^2^	0.21		0.15	
F	49.63**		22.67**	
N	750		674	

*CI* confidence interval; *p < 0.05. **p < 0.01

## Discussion

### Factors associated with physical fitness in boys and girls

This study investigated parental health-related behaviours and parental confidence in their ability to successfully encourage their children to be physically active. Results showed a significant effect of parental characteristics on PF in both, boys and girls. In accordance with previous research (Karppanen et al. 2012) this study identified especially mothers’ health-related behaviours and confidence in their ability to successfully encourage their children as important for children’s PF. This finding confirms the results of Bois and Sarrazin (2005) who emphasised the role of mothers as important socialisation agents due to their high involvement in day-to-day activity choices of children. Since mothers generally spend more time with their children compared to fathers (Sayer et al. 2004), it can be assumed that the mother’s attitudes toward a healthy and active lifestyle primarily influence children’s activity behaviour. Therefore, considering the close connection between PA and PF (Dencker et al. 2006) it can be assumed that especially mothers’ health-related behaviours and their confidence to successfully encourage their children to be active lead to a higher fitness level in children.

This study also showed that maternal smoking is negatively related to endurance performance in boys and girls. This confirms an early result of Burke et al. (1998) who identified parental smoking in a study of 800 10-to 12-year-olds as a predictor for lower PA and higher screen media consumption. They also concluded that poor health-related behaviours, including parental smoking, especially maternal smoking, are likely to influence children’s health behaviours. Further, it has been shown that passive smoking is an independent risk factor for respiratory symptoms (Larsson et al. 2003). In this study, it was shown that smoking parents are less likely to encourage their children to be active, which may potentially be based on poor health-related behaviours and attitudes. This lack of support and encouragement may also lead to lower aerobic capacity in their children. Furthermore, it can be assumed that passively smoking children display lower aerobic performances because of health reasons, especially respiratory illnesses (Larsson et al. 2003). Since mothers spend more time with their children, their smoking behaviour has the possibility to influence their children’s PF.

In addition to smoking, parental BMI has been investigated in order to find connections to children’s PA and PF (Magnusson et al. 2008). In agreement with these authors, this study showed no association between parental BMI and children’s PF in the final model. Magnusson et al. (2008) also concluded that other personal and health-related parental factors are more closely related to PF in children than solely their BMI. It seems that parental weight is less related to children’s activity behaviour and therefore PF than parental encouragement and support to be active.

Children’s PF, however, is highly associated with their own BMI. It is known that overweight and obese children have lower fitness levels than their lean counterparts (Grund et al. 2000). Karppanen et al. (2012) showed a negative relationship between overweight and impaired performance requiring balance and endurance in both genders. These authors also reported lower PA levels in overweight boys compared to boys with normal weight.

However, not only parental and children’s characteristics affect childhood PF; the present study also shows that socioeconomic variables such as family income and parental education level are associated with children’s PF, too. Family income affects boys’ coordination and girls’ aerobic capacity, whereas education only affects girls’ coordination. Even though the variables differ slightly for girls and boys, the results are consistent with previous research (Magnusson et al. 2008), which identified socioeconomic variables as significant predictors of children’s PF. A large German study (*n* = 2574) in children and adolescents showed that 6 to 9 year olds with a lower SES background had lower levels of PF than children with a higher SES background (Lämmle et al. 2012). Furthermore, using objective measurements to assess fitness levels of 488 9-year-oldchildren, an Icelandic study identified background variables such as areas of living and fathers’ income as important predictors of fitness (Magnusson et al. 2008). Therefore, it can be suggested that children with a higher socioeconomic background may receive more logistic and financial support as well as more encouragement to be active.

### Factors associated with physical fitness in boys

This study also showed differential influences on boys and girls. It was shown that mothers’ confidence in their ability to encourage and support their children to be physically active is related to both endurance and coordination in boys. In accordance with Trost and Loprinzi (2011), who showed in a recent review that parental support was consistently positively associated with childhood activity, it can be assumed that parental, especially maternal, support such as signing boys up for sports programmes, providing reinforcement for PA engagement and transport to sports facilities is important for PA.

Results showed that parental support was consistently positively and significantly associated with child activity. Since high-intensityphysical activity is one of the main determinants for PF (Ortega et al. 2008), it can be concluded that mothers’ confidence in their ability to encourage and support their children to more PA leads to an increase in PF.

Besides, the present study shows that endurance and coordination in boys are not only affected by maternal confidence to encourage being physically active, but also their PA behaviour. This confirms the result of Fuemmeler et al. (2011), who identified parents serving as role models for children’s PA. In accordance to Trost et al. (2003) it can be assumed that parents with an active lifestyle and knowledge of the importance of PA are more likely to support their children to be physically active. Thereby again, especially mothers’ PA seems to be related to children’s PA behaviour (Karppanen et al. 2012). Since mothers are known to spend more time with their children (Sayer et al. 2004), it can be assumed that they are more likely to encourage their children to be more physically active. In adolescents, relatives’ PA engagement has been shown to be associated with PF (Martín-Matillas et al. 2012). In a study with 3,288 12.5–17.5-year-old adolescents (48 % boys), it was demonstrated that fathers’, mothers’, brothers’ and best friends’ activity behaviours were positively related to cardiorespiratory fitness. In children, Cleland et al. (2005) identified parental PA as positively associated with their cardiorespiratory fitness, as assessed by two fitness tests—a 1-mile run/walk and cycle ergometry. On the other hand, Trost et al. (2003) identified parental support such as transportation to sporting and fitness activities rather than being a role model as related factors for activity levels in children. These complementary although inconsistent results may be due to the different methods of data collection and a lack of consideration of covariates on PF.

### Factors associated with physical fitness in girls

The previously mentioned factors were associated with boys’ PF, but this study also identified aspects that mainly influenced girls’ PF. Mothers’ confidence in their ability to encourage their children to be physically active affected boys’ endurance and coordination. In girls, however, only coordination was affected by mothers’ confidence in their ability to encourage their girls to engage in sufficient PA. Again, it can be assumed that especially mothers’ support seems to have a great effect on girls’ PF. Also, girls are known to choose more coordinating activities such as skipping or dancing in their leisure time (Nielsen et al. 2011). Looking at numbers of female members in German sports clubs, they mostly favour coordination promoting sports such as gymnastics, horse riding and dancing (Breuer 2009). Since coordination-promotingsports seem to be preferred by girls, it can be suggested that mothers encourage and mainly support their children’s interests and overall PA without selecting specific sports for their children. This may—then more due to the child’s interest—lead to girls engaging in more coordination than endurance-promotingsports. However, this does not fully explain this circumstance since girls show no better performance in lateral jumping than boys. Therefore, and to gain more insight, more research is required.

Endurance in girls, however, was affected by family income and migration. Whilst research on the impact of migration on PA and particularly on PF in children is rare, it is known that especially girls with a migration background show a lack of PA (Jekauc et al. 2012). Recent research shows that girls with a migration background are more than twice as likely not to comply with PA guidelines compared with girls without a migration background (Jekauc et al. 2012). Against this context, it can be assumed that ethnic differences and different values also have an effect on PF levels in girls. In this field, more research is required.

Further, this study showed an effect of parental education on girls’ coordination. The evidence regarding the effect of parental education on children’s PF is rare. However, our results are in line with a current German study that found a positive association between parental education and fitness in children (Finger et al. 2014). Investigating 5,251 participants at the age of 11–17 years, it was shown that girls of parents with high socioeconomic status are more physically active in their leisure time, whereby especially parental education seems to play a crucial role. Based on these results it can be assumed that well-educatedparents know the importance of PA and PF in children and therefore are more supportive and encouraging in children’s PA. In addition, Gustafson and Rhodes (2006) identified sex-dependant differences in parental support to be active. Since parents seem to perceive a higher importance of PA in boys (Trost et al. 2003), it can be hypothesised that boys are encouraged to be active through all parental education levels by different parental motives. In girls especially health-related parental motives and therefore the parental education level could be the crucial factor.

This study provides valuable insights into the association of parental health behaviour and children’s PF. However, several aspects should be considered when interpreting these results. One limiting factor of this study is the cross-sectionaldesign, which does not allow conclusions about the direction of these associations. Also, the use of subjective measurements to assess parental health behaviours and confidence in their ability to encourage their children is a limitation of this study since some of the questions might have been answered in a socially desired fashion. Additionally, it should be noted that the questionnaire was mainly completed by mothers, whereby it is not known if mothers answered the questions on the father’s health behaviour with or without consulting them. Considering that this study was conducted in southwest Germany, and only children whose teachers agreed to participate were involved, the findings are not representative for the whole of Germany. Due to the large sample size, the high response rate (76 %) and the objective measurement of children’s PF and body composition, however, this study adds important information about the context of parental health behaviour and children’s PF.

## Conclusion

In a large cross-sectionalsample in southern Germany, this study showed that parental health-related behaviours and their confidence in their ability to encourage their children to be physically active affects children’s PF. Although parental influence on children’s PF differed slightly in girls and boys, it could be shown that especially mothers play an important role in children’s PF. Particularly their perceived ability to encourage their children to be active is closely connected to children’s PF. This strongly supports the aspect of including parents in the development of health promotion and obesity prevention programmes in children. Concepts that show parents how to encourage their children to be active should also be considered and future research should include measurements of parental encouragement and their confidence in their ability to encourage their children as well as additional influencing factors such as nutrition habits and media consumption to understand the influence of parental health-related behaviours on children’s PF in an overall context.
